# Structure of metaraminol hydrogen tartrate from synchrotron X-ray data and density functional theory calculations

**DOI:** 10.1107/S2056989026009497

**Published:** 2026-09-15

**Authors:** Jacob K. Salazar, James A Kaduk, Anja Dosen, Thomas N. Blanton

**Affiliations:** a North Central College, Department of Chemistry 131 S Loomis St Naperville IL 60540 USA; b North Central College, Department of Physics 131 S Loomis St Naperville IL 60540 USA; c ICDD, 12 Campus Blvd., Newtown Square PA 19073-3273, USA; University of Aberdeen, United Kingdom

**Keywords:** powder diffraction, aramine, Rietveld refinement, density functional theory, crystal structure

## Abstract

The crystal structure of the title salt, C_9_H_14_NO_2_^+^·HC_4_H_4_O_6_^−^, has been solved and refined using synchrotron X-ray powder diffraction data and optimized using density functional theory techniques. The crystal structure consists of alternating hydro­phobic and hydro­philic layers lying parallel to the *ab* plane. Hydrogen bonds link the cations and anions in the hydro­philic layers. The anions are linked by very strong charge-assisted O—H⋯O hydrogen bonds into chains propagating along the *a*-axis direction. Each H atom of the protonated N atom of the cation acts as a donor in at least one N—H⋯O hydrogen bond to an anion.

## Abstract 2

The crystal structure of metaraminol hydrogen tartrate has been solved and refined using synchrotron X-ray powder diffraction data and optimized using density functional theory techniques.

## 1. Chemical context

Metaraminol hydrogen tartrate, C_9_H_14_NO_2_^+^·HC_4_H_4_O_6_^−^ (**I**), also known as metaraminol bitartrate, and marketed as Aramine (obsolete), Pressonex and others in various countries, is used to treat hypotension (low blood pressure), particularly associated with patients receiving spinal anesthesia (Grauslyte *et al.*, 2022 ▸). The systematic name (CAS Registry Number 33402-03-8) is 3-[(1*R*,2*S*)-2-azaniumyl-1-hy­droxy­prop­yl]phenol (2*S*,3*S*)-hydrogen 2,3-di­hydroxy­but­ane­dioate.
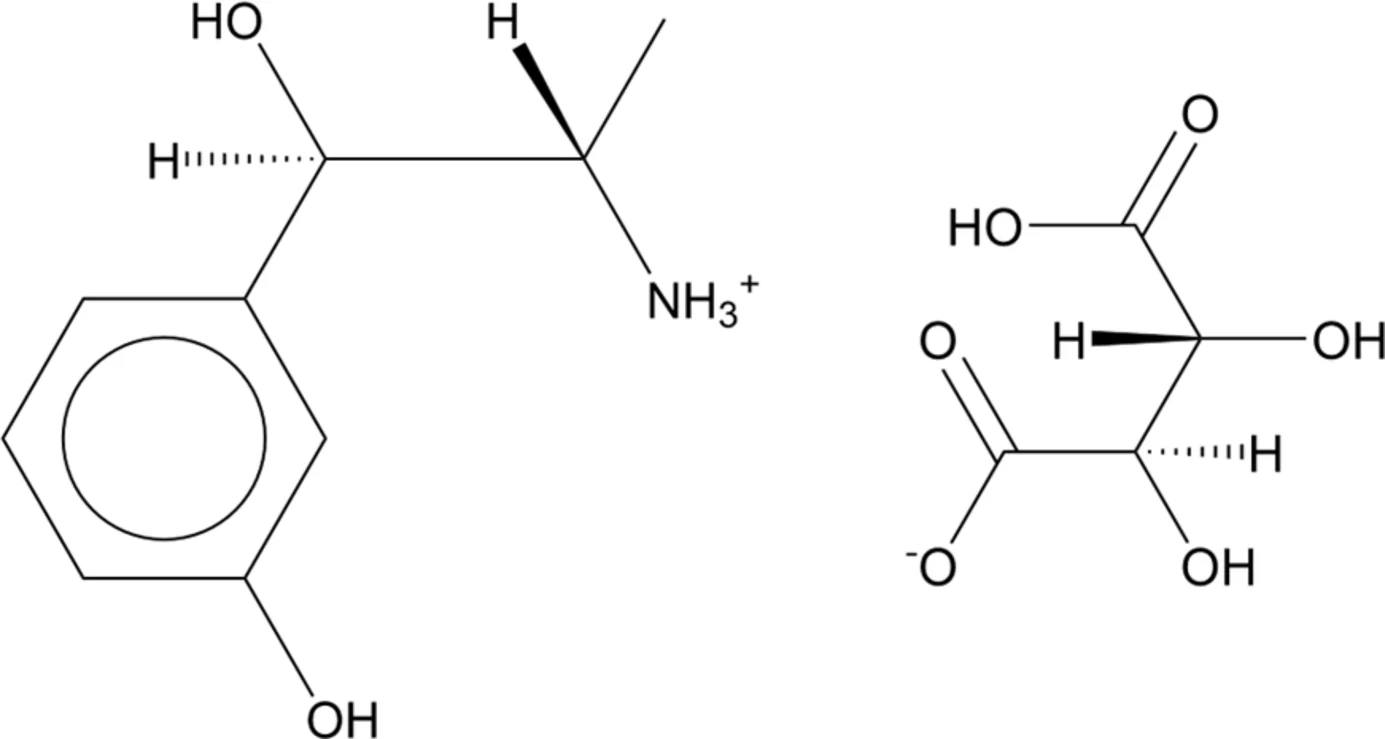


This work was carried out as part of a project (Kaduk *et al.*, 2014 ▸) to determine the crystal structures of large-volume commercial pharmaceuticals, and includes high-quality powder diffraction data for them in the Powder Diffraction File (Kabekkodu *et al.*, 2024 ▸).

## 2. Structural commentary

**Figure 1:**
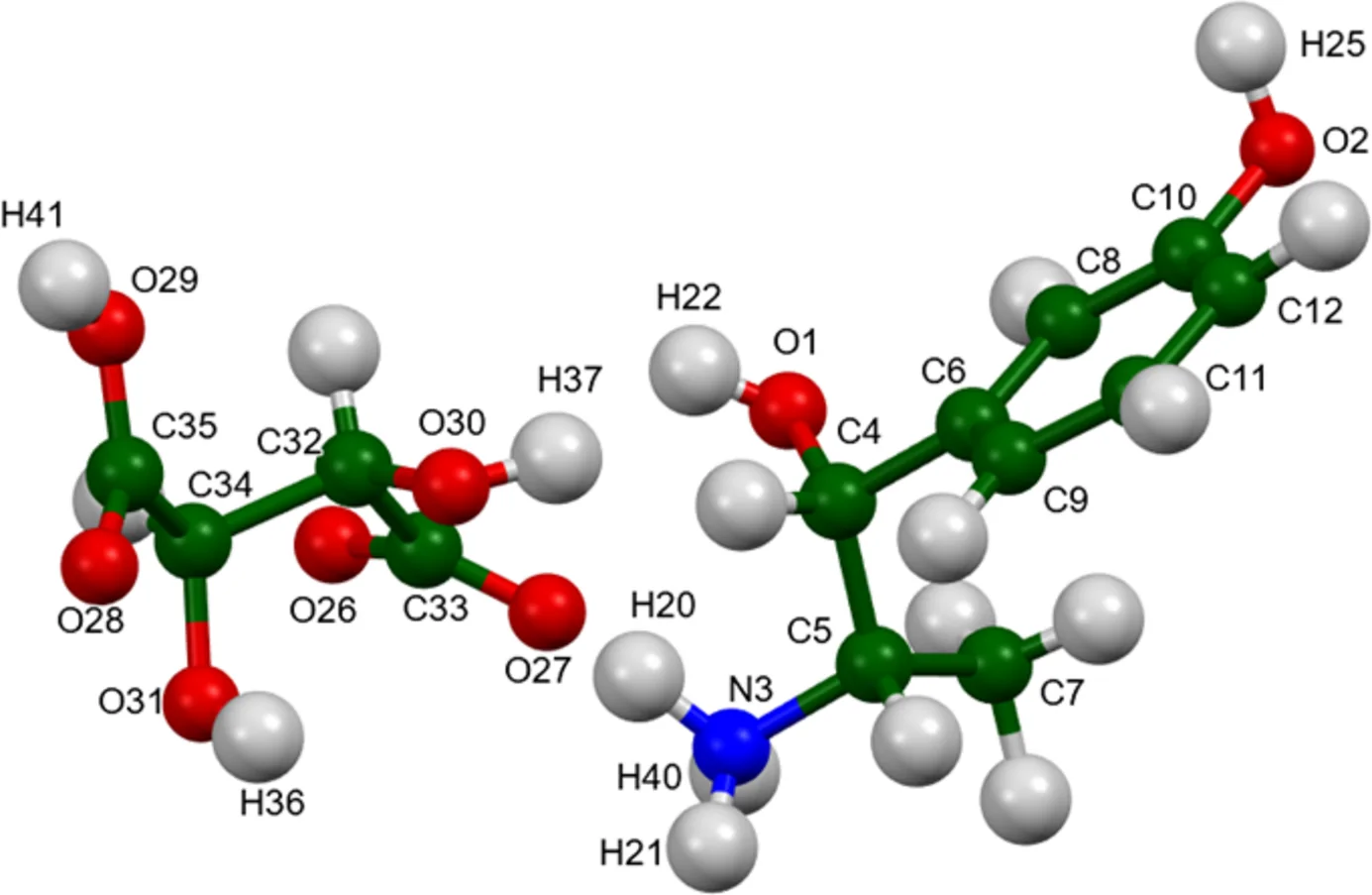
The asymmetric unit of (**I**), with the atom numbering. The atoms are represented by 50% probability spheroids.

**Figure 2:**
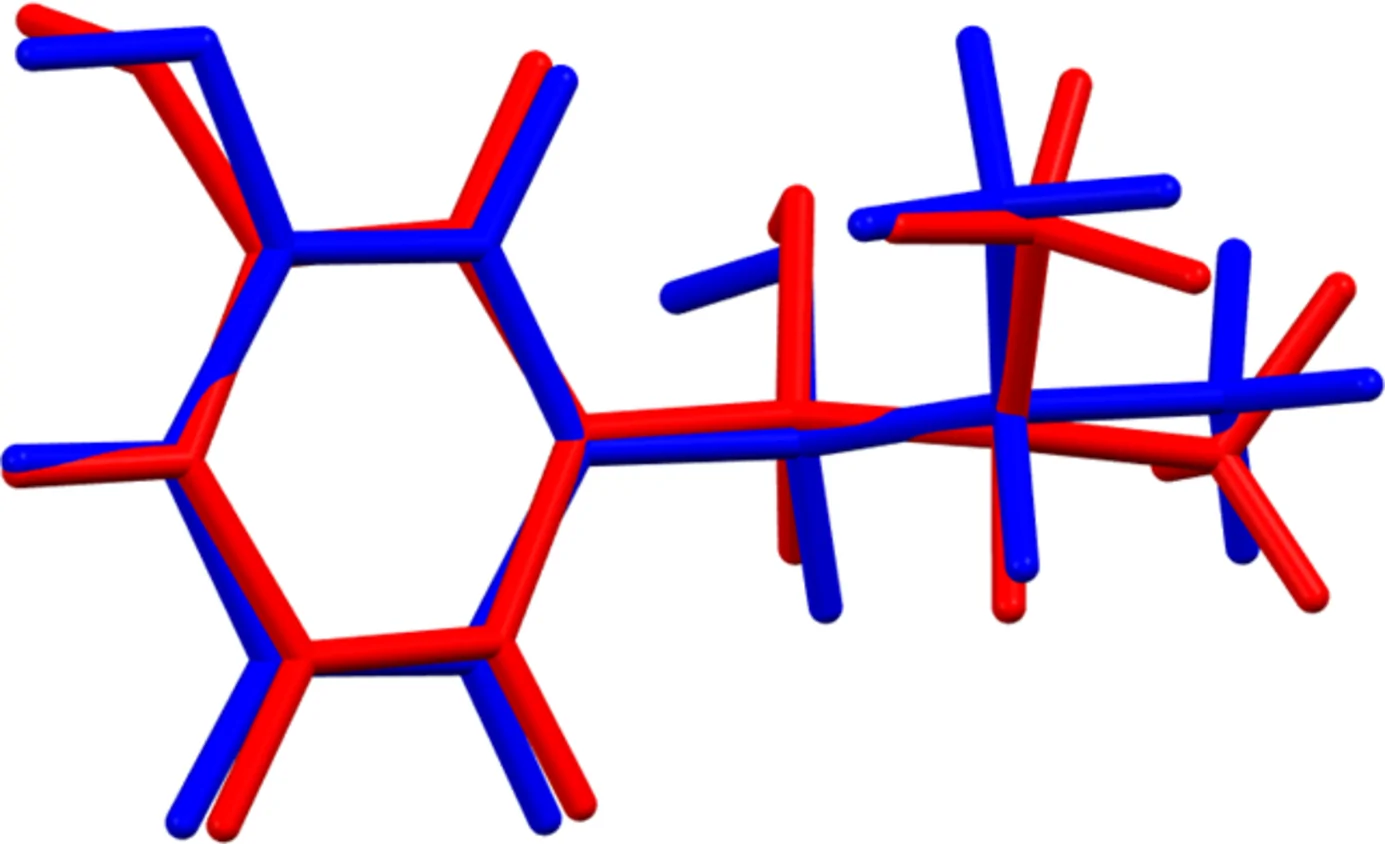
Comparison of the refined structure of the metaraminol cation in (**I**) (red) to the *VASP*-optimized structure (blue). The comparison was generated using the *Mercury* calculate/mol­ecule overlay tool; the r.m.s. difference is 0.235 Å.

The asymmetric unit of (**I**) is illustrated in Fig. 1 ▸ and consists of one C_9_H_14_NO_2_^+^ cation and one HC_4_H_4_O_6_^−^ anion in space group *P*2_1_2_1_2_1_. The root-mean-square difference of the non-H atoms in the Rietveld-refined and *VASP*-optimized structures of (**I**), calculated using the *Mercury* (Macrae *et al.*, 2020 ▸) CSD-Materials/search/crystal packing similarity tool is 0.325 Å. The root-mean-square Cartesian displacements of the non-H atoms in the refined and optimized structures of the cation and anion, calculated using the *Mercury* calculate/mol­ecule overlay tool, are 0.235 and 0.167 Å, respectively (Figs. 2 ▸ and 3 ▸). The agreements are within the normal range for correct structures (van de Streek & Neumann, 2014 ▸). The remaining discussion will emphasize the *VASP*-optimized structure.

Almost all of the bond distances, bond angles, and torsion angles fall within the normal ranges indicated by a *Mercury* Mogul geometry check (Macrae *et al.*, 2020 ▸). Only the C7—C5—C4 bond angle of 117.1° [average = 114.1 (9)°; *Z*-score = 3.4] is flagged as unusual. The standard uncertainty on the average is very small, inflating the *Z*-score, so this angle is not of concern. The conformation about the C6—C4—C5—N3 torsion angle is *trans* (179.2°), and falls within the normal distribution. The torsion angles involving rotation about the C4—C6 bond (such as 77.9° for C5—C4—C6—C8) fall within the typical broad ranges for similar torsion angles. The tartrate anion is in the *trans* conformation, as indicated by the C35—C34—C32—C33 torsion angle of −177.2°.

Quantum chemical geometry optimizations of the isolated metaraminol cation and bitartrate anion (DFT/B3LYP/6-31G*/water) using *Spartan ’24* (Wavefunction, 2025 ▸) indicated that both the cation and anion were geometrically (r.m.s. difference = 0.277 and 0.173 Å, respectively) similar to local minima, but energetically different (6.1 and 21.2 kcal mol^−1^, respectively). The global minimum-energy conformations were found to be more compact (featuring intra­molecular hydrogen bonds), showing that inter­molecular inter­actions are important in determining the solid-state conformations of these species in (**I**).

## 3. Supra­molecular features

**Figure 4:**
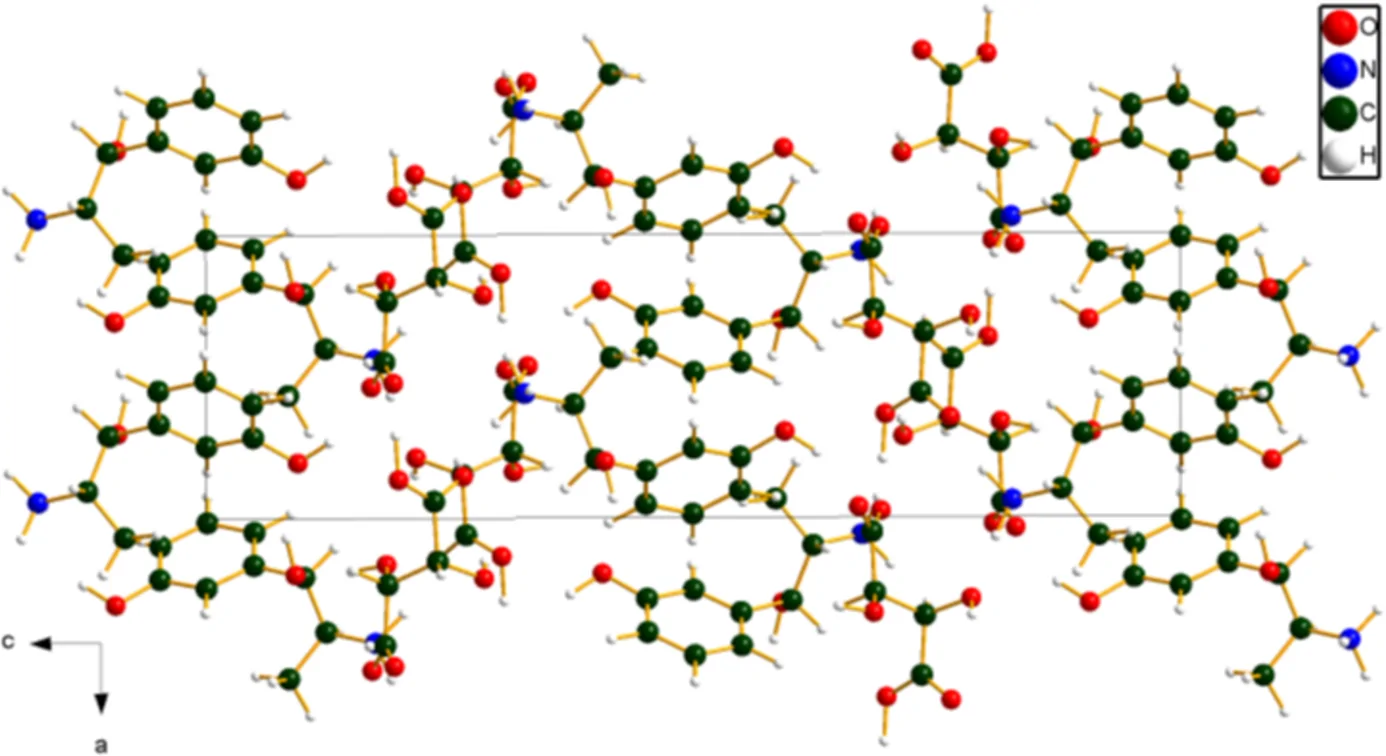
The crystal structure of (**I**), viewed down the *b*-axis direction.

The extended structure of (**I**) (Fig. 4 ▸) consists of alternating hydro­phobic and hydro­philic layers lying parallel to the *ab* plane. Hydrogen bonds (discussed below) link the cations and anions in the hydro­philic layers. The *Mercury* aromatics analyser indicated one strong (*d* = 4.97 Å) inter­action between the phenyl rings of the cations, and two moderate (*d* = 6.15 Å) inter­actions. The mean plane of the phenyl ring in the asymmetric unit is approximately (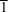
11).

Analysis of the contributions to the total crystal energy of the structure using the Forcite module of *Materials Studio* (Dassault Systèmes, 2025 ▸) indicated that bond, angle, and torsion distortion terms contribute significantly to the intra­molecular energy. The inter­molecular energy is small, and is dominated by van der Waals attractions, which in this force field-based analysis includes hydrogen bonds. The hydrogen bonds are better discussed using the results of the DFT calculation.

Hydrogen bonds (Table 1 ▸) are prominent in the structure. The anions are linked by very strong charge-assisted O29—H41⋯O26 hydrogen bonds into chains propagating along the *a*-axis direction. The O29—H41 single bond has a Mulliken overlap population of 0.175 *e*, with a bond energy [calculated using the correlation of Rammohan & Kaduk (2018 ▸)] of 23.9 kcal mol^−1^. The H41⋯O26 hydrogen bond is only slightly weaker, with an overlap population of 0.117 *e* and a bond energy of 18.7 kcal mol^−1^. Both alcoholic hydroxyl groups of the hydrogen tartrate anion act as donors in O—H⋯O hydrogen bonds, one to another anion and one to a cation.

Each H atom of the protonated –N3H_3_^+^ grouping of the cation acts as a donor in at least one N—H⋯O hydrogen bond to an anion. The energies of these hydrogen bonds were calculated using the correlation of Wheatley & Kaduk (2019 ▸). One hydroxyl group of the cation forms an O—H⋯O hydrogen bond to another cation, while the other bonds to the anion. Several C—H⋯O hydrogen bonds (from both the cation and anion) also contribute to the cohesion of the structure.

**Figure 5:**
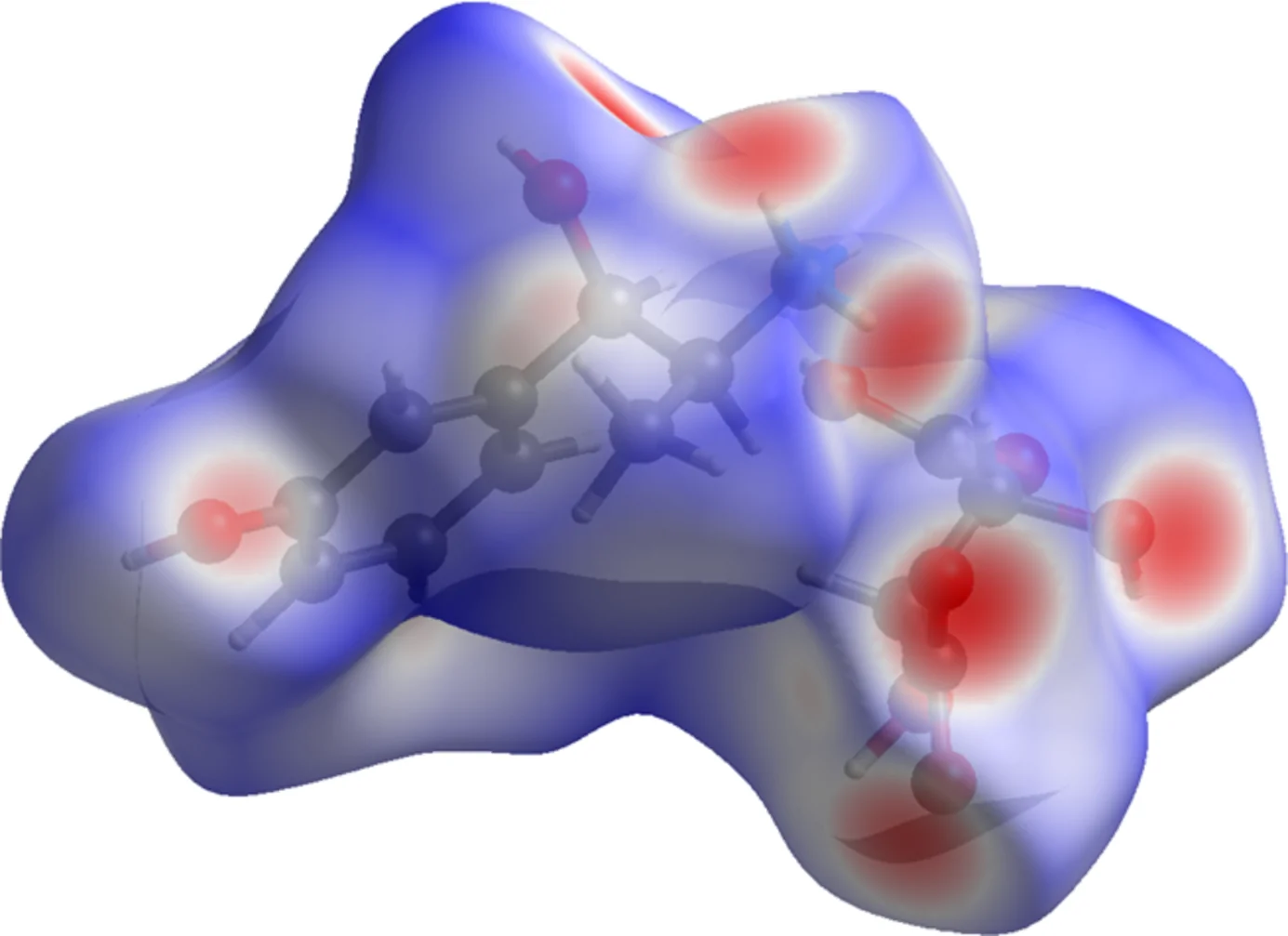
The Hirshfeld surface of (**I**). Inter­molecular contacts longer than the sums of the van der Waals radii are colored blue, and contacts shorter than the sums of the radii are colored red. Contacts equal to the sums of radii are white.

The volume enclosed by the Hirshfeld surface of (**I**) (Fig. 5 ▸; Spackman *et al.*, 2021 ▸) is 366.73 Å^3^, or 98.13% of 1/4 of the unit-cell volume. The packing density is thus typical. The close contacts (red in Fig. 5 ▸) correspond to the hydrogen bonds noted above. The volume per non-hydrogen atom is slightly smaller than normal, at 17.0 Å^3^.

The Bravais–Friedel–Donnay–Harker (Bravais, 1866 ▸; Friedel, 1907 ▸; Donnay & Harker, 1937 ▸) algorithm suggests that we might expect platy morphology for (**I**), with {001} as the principal faces. A second-order spherical harmonic model for preferred orientation was included. The texture index was 1.001, indicating that the preferred orientation was negligible in this rotated capillary specimen.

## 4. Database survey

A process for preparing metaraminol bitartrate is claimed in US Patent 10,087,136 B2 (Brenna *et al.*, 2018 ▸; Laboratori Alchemia S.r.l.), which includes NMR data, but no X-ray diffraction data, is provided. A synthesis method for metaraminol bitartrate is also claimed in Chinese Patent CN 103739504A (Pu *et al.*, 2013 ▸; Guangzhou Person Pharmaceutical Co., Ltd.). We are unaware of any published diffraction data for metaraminol bitartrate.

A reduced cell search in the Cambridge Structural Database (CSD version 2026.1.0; Groom *et al.*, 2016 ▸), combined with the chemistry C, H, N, and O only, yielded 31 hits, but no structures of metaraminol or its derivatives.

## 5. Synthesis and crystallization

Metaraminolhydrogen tartrate is a commercial reagent, purchased from TargetMol (Batch #118859), and was used as received.

## 6. Refinement

Crystal data, data collection and structure refinement details are summarized in Table 2 ▸. The white powder was packed into a 1.5 mm diameter Kapton capillary, and rotated during the measurement at ∼50 Hz. The powder pattern was measured at 295 K at beam line 11-BM (Lee *et al.*, 2008 ▸; Wang *et al.*, 2008 ▸; Antao *et al.*, 2008 ▸) of the Advanced Photon Source at Argonne National Laboratory using a wavelength of 0.4687342 Å from 0.5–50° 2θ with a step size of 0.001° and a counting time of 0.1 sec step^−1^. The high-resolution powder diffraction data were collected using twelve silicon crystal analyzers that allow for high angular resolution, high precision, and accurate peak positions. A mixture of silicon (NIST SRM 640c) and alumina (NIST SRM 676a) standards (ratio Al_2_O_3_:Si = 2:1 by weight) was used to calibrate the instrument and refine the monochromatic wavelength used in the experiment.

The pattern was indexed on a primitive ortho­rhom­bic unit cell with *a* = 7.19584, *b* = 8.42890, *c* = 24.73316 Å, *V* = 1500.14 Å^3^, and *Z* = 4 using *JADE Pro* (MDI, 2025 ▸). The suggested space group was *P*2_1_2_1_2_1_, which was confirmed by the successful solution and refinement of the structure.

The mol­ecular structure of metaraminol was downloaded from PubChem (Kim *et al.*, 2023 ▸) as Conformer3D_COMPOUND_CID_5906.sdf. It was converted to a *.mol2 file using *Mercury* (Macrae *et al.*, 2020 ▸), and to a Fenske–Hall *Z*-matrix using *Open Babel* (O’Boyle *et al.*, 2011 ▸). The tartrate anion model was taken from our structure of eliglustat hemitartrate (Kaduk *et al.*, 2026 ▸). The structure was solved using Monte Carlo simulated annealing techniques as implemented in *DASH* (David *et al.*, 2006 ▸), *EXPO2014* (Altomare *et al.*, 2013 ▸), and *FOX* (Favre-Nicolin & Černý, 2002 ▸). All three programs yielded essentially the same structure. The *EXPO2014* model was chosen for refinement.

Atom H40 was added to protonate N3 using *Materials Studio* (Dassault Systèmes, 2025 ▸). Analysis of O⋯O distances for potential hydrogen bonds suggested that O29 was protonated (O29⋯O26 = 2.39 Å), so H41 was added 0.90 Å from O29 on the O29⋯O26 vector using *Materials Studio*.

Rietveld refinement was carried out using *GSAS-II* (Toby & Von Dreele, 2013 ▸). Only the 1.5–28.0° portion of the pattern was included in the refinement (*d*_min_ = 0.969 Å). All non-H bond distances and angles were subjected to restraints, based on a *Mercury*/Mogul Geometry Check (Sykes *et al.*, 2011 ▸; Bruno *et al.*, 2004 ▸). The Mogul average and standard deviation for each qu­antity were used as the restraint parameters. The phenyl ring was restrained to be planar. The restraints contributed 1.5% to the overall *χ^2^*. The hydrogen atoms were included in calculated positions, which were recalculated during the refinement using *Materials Studio* (Dassault Systèmes, 2025 ▸). The *U*_iso_ of the non-H atoms were grouped by chemical similarity. The *U*_iso_ of the H atoms were fixed at 1.2× the *U*_iso_ of the heavy atom to which they are attached. The peak profiles were described using a uniaxial microstrain model, with [001] as the unique axis. The background was modeled using a six-term shifted Chebyshev polynomial, with a peak at 6.00° to model the scattering from the Kapton capillary and any amorphous component of the sample.

**Figure 6:**
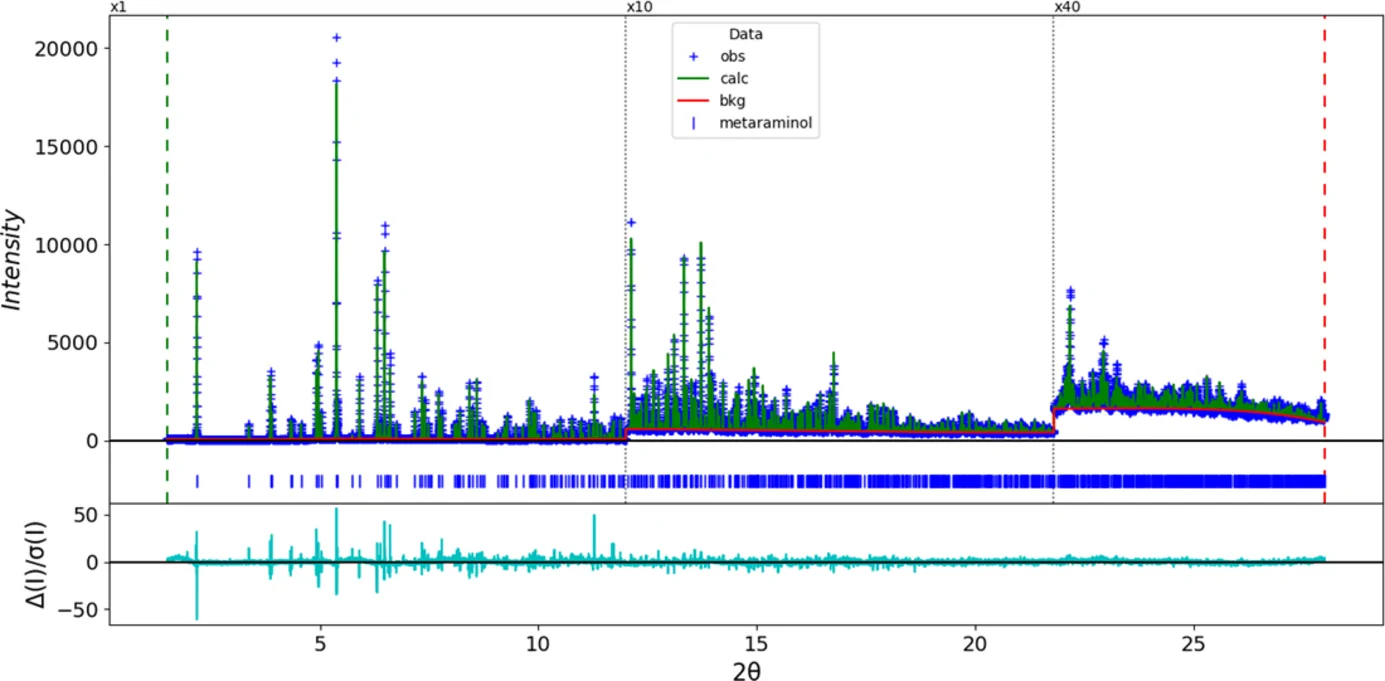
The Rietveld plot for (**I**). The blue crosses represent the observed data points, and the green line is the calculated pattern. The cyan curve is the normalized error plot, and the red line is the background curve. The blue tick marks indicate the peak positions. The vertical scale has been multiplied by a factor of 10× for 2θ > 12.0°, and by a factor of 40× for 2θ > 21.8°.

The final refinement of 85 variables using 26,501 observations and 50 restraints yielded the residuals *R*_wp_ = 0.1055 and GOF = 2.74. The largest peak (0.24 Å from N3) and hole (0.82 Å from C6) in the difference Fourier map were calculated to be 0.56 (11) and −0.45 (11) e Å^−3^, respectively. The final Rietveld plot is shown in Fig. 6 ▸. The largest features in the normalized error plot are in the shapes of some of the strong low-angle peaks.

The crystal structure of (**I**) was optimized (fixed experimental unit cell) with density functional theory techniques using *VASP* (Kresse & Furthmüller, 1996 ▸) through the *MedeA* graphical inter­face (Materials Design, 2024 ▸). The calculation was carried out on 32 cores of a 144-core (768 GB memory) HPE Superdome Flex 280 Linux server at North Central College. The calculation used the GGA-PBE functional, a plane wave cutoff energy of 400.0 eV, and a *k*-point spacing of 0.5 Å^−1^ leading to a 2 × 2 × 1 mesh, and took ∼8.5 h. Single-point density functional theory calculations (fixed experimental cell) and population analysis were carried out using *CRYSTAL23* (Erba *et al.*, 2023 ▸). The basis sets for the H, C, N and O atoms in the calculation were those of Gatti *et al.* (1994 ▸). The calculations were run on a 3.5 GHz PC using 8 *k*-points and the B3LYP functional, and took ∼1.9 h. The powder pattern of (**I**) has been submitted to ICDD for inclusion in the Powder Diffraction File.>

## Supplementary Material

Crystal structure: contains datablock(s) I, I_VASP. DOI: 10.1107/S2056989026009497/hb8243sup1.cif

CCDC references: 2587069, 2587068

Additional supporting information:  crystallographic information; 3D view; checkCIF report

## Figures and Tables

**Figure 3:**
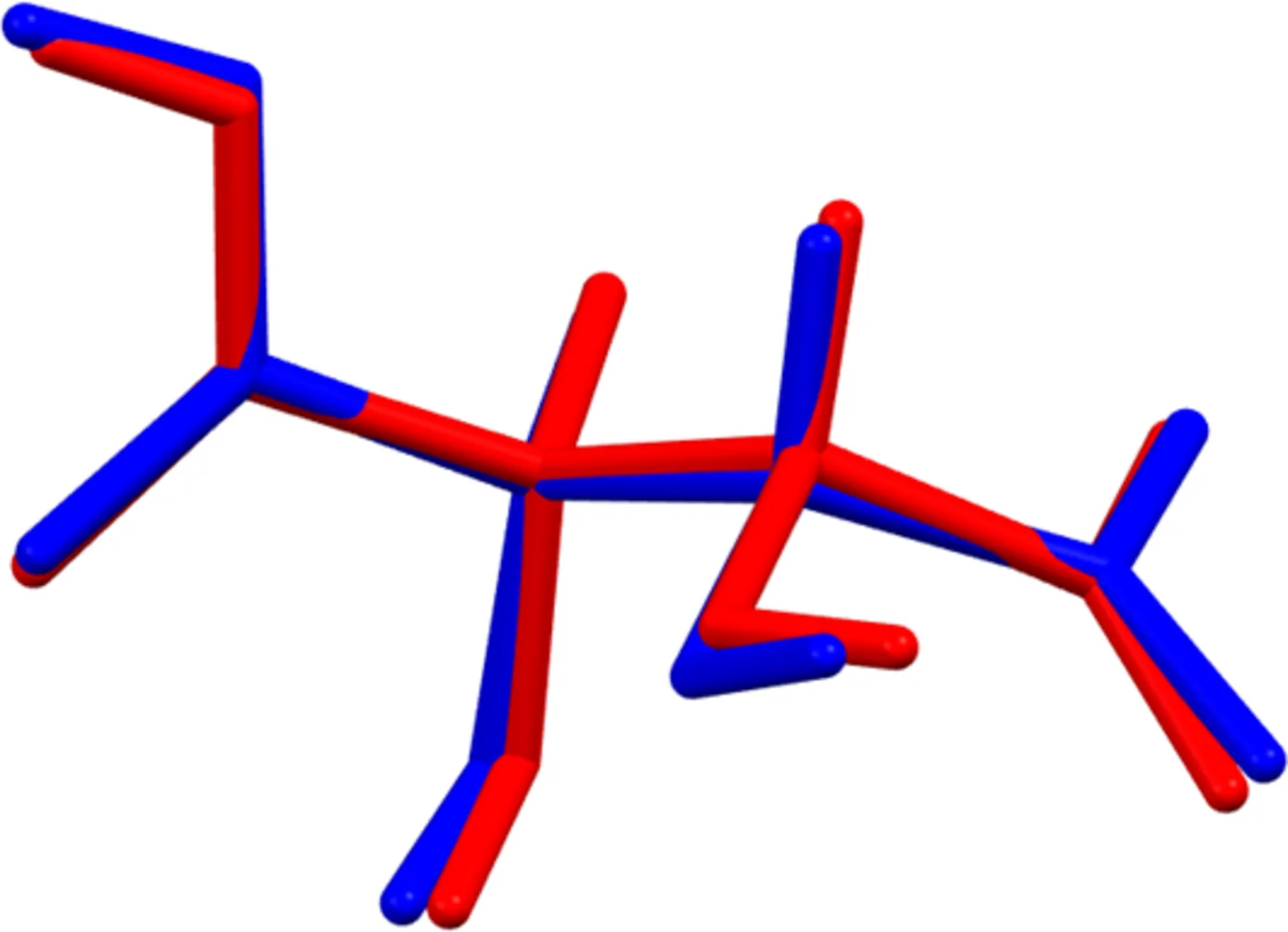
Comparison of the refined structure of the bitartrate anion in (**I**) (red) to the *VASP*-optimized structure (blue). The comparison was generated using the *Mercury* calculate/mol­ecule overlay tool; the r.m.s. difference is 0.167 Å.

**Table 1:** Hydrogen-bond geometry (Å, °)

*D*—H⋯*A*	*D*—H	H⋯*A*	*D*⋯*A*	*D*—H⋯*A*
O29—H41⋯O26^i^	1.14	1.33	2.464	173
O30—H37⋯O1	0.99	1.70	2.686	169
O31—H36⋯O29^ii^	0.99	1.95	2.885	156
O31—H36⋯O28	0.99	2.36	2.644	96
N3—H40⋯O31^iii^	1.05	1.72	2.762	172
N3—H21⋯O28^ii^	1.05	1.69	2.718	166
N3—H20⋯O27	1.05	1.78	2.796	161
N3—H20⋯O30	1.05	2.50	3.111	116
O2—H25⋯O27^iv^	1.00	1.76	2.709	157
O1—H22⋯O2^iv^	0.98	2.19	3.113	155
C5—H14⋯O26	1.10	2.40	3.364	145
C12—H24⋯O26	1.09	2.72	3.758	154
C4—H13⋯O28	1.11	2.61	3.410	128
C9—H19⋯O29	1.09	2.95	4.033	171

Symmetry codes: (i) 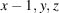; (ii) 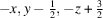; (iii) 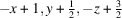; (iv) 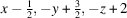.

**Table 2:** Experimental details

	(I)
Crystal data	
Chemical formula	C_9_H_14_NO_2_^+^·C_4_H_5_O_6_^−^
*M*_r_	317.29
Crystal system, space group	Orthorhombic, *P*2_1_2_1_2_1_
Temperature (K)	295
*a*, *b*, *c* (Å)	7.190512 (12), 8.421634 (17), 24.69323 (5)
*V* (Å^3^)	1495.32 (1)
*Z*	4
Radiation type	Synchrotron, λ = 0.46873 Å
μ (mm^−1^)	0.001
Specimen shape, size (mm)	Cylinder, 2.0 × 1.5

Data collection	
Diffractometer	11-BM, APS
Specimen mounting	Kapton capillary
Data collection mode	Transmission
Scan method	Step
2θ values (°)	2θ_min_ = 0.510, 2θ_max_ = 49.995, 2θ_step_ = 0.001

Refinement	
*R* factors and goodness of fit	*R*_p_ = 0.090, *R*_wp_ = 0.105, *R*_exp_ = 0.039, *R*(*F*^2^) = 0.09024, χ^2^ = 7.530
No. of parameters	85
No. of restraints	50
(Δ/σ)_max_	2.873

Computer programs: *GSAS-II* (Toby & Von Dreele, 2013 ▸) and *DIAMOND* (Crystal Impact, 2025 ▸).

## supplementary crystallographic information

### 3-[(1R,2S)-2-Azaniumyl-1-hydroxypropyl]phenol (2S,3S)-hydrogen 2,3-dihydroxybutanedioate (I) Crystal data

**Table:**

C_9_H_14_NO_2_^+^·C_4_H_5_O_6_^−^	*Z* = 4
*M**_r_* = 317.29	*D*_x_ = 1.409 Mg m^−^^3^
Orthorhombic, *P*2_1_2_1_2_1_	Synchrotron radiation, λ = 0.46873 Å
*a* = 7.190512 (12) Å	µ = 0.001 mm^−^^1^
*b* = 8.421634 (17) Å	*T* = 295 K
*c* = 24.69323 (5) Å	cylinder, 2.0 × 1.5 mm
*V* = 1495.32 (1) Å^3^	

### 3-[(1R,2S)-2-Azaniumyl-1-hydroxypropyl]phenol (2S,3S)-hydrogen 2,3-dihydroxybutanedioate (I) Data collection

**Table:**

11-BM, APS diffractometer	Scan method: step
Specimen mounting: Kapton capillary	2θ_min_ = 0.510°, 2θ_max_ = 49.995°, 2θ_step_ = 0.001°
Data collection mode: transmission	

### 3-[(1R,2S)-2-Azaniumyl-1-hydroxypropyl]phenol (2S,3S)-hydrogen 2,3-dihydroxybutanedioate (I) Refinement

**Table:**

Least-squares matrix: full	85 parameters
*R*_p_ = 0.090	50 restraints
*R*_wp_ = 0.105	19 constraints
*R*_exp_ = 0.039	Weighting scheme based on measured s.u.'s
*R*(*F*^2^) = 0.09024	(Δ/σ)_max_ = 2.873
49486 data points	Background function: Background function: "chebyschev-1" function with 6 terms: 44.39(10), -9.67(15), -6.44(12), -0.91(18), 1.31(17), -5.45(13), Background peak parameters: pos, int, sig, gam: 5.974(12), 1.053(24)e4, 1.09(3)e4, 0.100,
Profile function: Finger-Cox-Jephcoat function parameters U, V, W, X, Y, SH/L: peak variance(Gauss) = Utan(Th)^2^+Vtan(Th)+W: peak HW(Lorentz) = X/cos(Th)+Ytan(Th); SH/L = S/L+H/L U, V, W in (centideg)^2^, X & Y in centideg 1.163, -0.126, 0.063, 0.000, 0.000, 0.002,	Preferred orientation correction: Simple spherical harmonic correction Order = 2 Coefficients: 0:0:C(2,0) = -0.0120; 0:0:C(2,2) = 0.0660

### 3-[(1R,2S)-2-Azaniumyl-1-hydroxypropyl]phenol (2S,3S)-hydrogen 2,3-dihydroxybutanedioate (I) Fractional atomic coordinates and isotropic or equivalent isotropic displacement parameters (Å^2^)

**Table:**

	*x*	*y*	*z*	*U*_iso_*/*U*_eq_	
O1	0.3342 (4)	0.8022 (3)	0.93215 (10)	0.0479 (6)*	
O2	0.3359 (4)	0.5307 (3)	1.10736 (10)	0.0445 (5)*	
N3	0.4468 (4)	0.6614 (4)	0.82720 (13)	0.0479 (6)*	
C4	0.2734 (5)	0.6488 (4)	0.91486 (13)	0.0479 (6)*	
C5	0.4270 (5)	0.5755 (4)	0.87984 (13)	0.0479 (6)*	
C6	0.2282 (5)	0.5511 (4)	0.96343 (12)	0.0445 (5)*	
C7	0.6176 (5)	0.5688 (5)	0.90906 (15)	0.0479 (6)*	
C8	0.3051 (6)	0.5873 (4)	1.01338 (14)	0.0445 (5)*	
C9	0.1121 (5)	0.4235 (4)	0.95835 (13)	0.0445 (5)*	
C10	0.2652 (5)	0.4938 (4)	1.05747 (11)	0.0445 (5)*	
C11	0.0749 (5)	0.3274 (4)	1.00251 (17)	0.0445 (5)*	
C12	0.1529 (6)	0.3636 (4)	1.05258 (13)	0.0445 (5)*	
H13	0.14930	0.66750	0.89080	0.0665*	
H14	0.38411	0.45668	0.86620	0.0665*	
H15	0.60500	0.49580	0.94340	0.0665*	
H16	0.70849	0.49856	0.88244	0.0665*	
H17	0.67350	0.67480	0.91330	0.0665*	
H18	0.40460	0.68710	1.01580	0.0655*	
H19	0.04630	0.38770	0.92000	0.0655*	
H20	0.34520	0.74740	0.82580	0.0665*	
H21	0.43010	0.59330	0.79430	0.0665*	
H22	0.24880	0.87450	0.92230	0.0665*	
H23	−0.02730	0.22950	1.00070	0.0655*	
H24	0.11220	0.29700	1.08940	0.0655*	
H25	0.25770	0.60010	1.12710	0.0655*	
O26	0.5203 (4)	1.2468 (3)	0.80511 (12)	0.0480 (4)*	
O27	0.5292 (3)	0.9921 (3)	0.83009 (11)	0.0480 (4)*	
O28	−0.1329 (3)	1.0797 (3)	0.73408 (11)	0.0480 (4)*	
O30	0.1607 (4)	0.9700 (3)	0.83668 (10)	0.0480 (4)*	
O31	0.2265 (3)	1.0825 (3)	0.72509 (10)	0.0480 (4)*	
C32	0.2339 (5)	1.1202 (4)	0.82380 (14)	0.0480 (4)*	
C33	0.4494 (4)	1.1141 (4)	0.82050 (19)	0.0480 (4)*	
C34	0.1555 (5)	1.1747 (4)	0.76783 (14)	0.0480 (4)*	
C35	−0.0592 (4)	1.1695 (5)	0.76762 (16)	0.0480 (4)*	
H36	0.18320	0.97650	0.72600	0.0675*	
H37	0.22630	0.92780	0.86820	0.0675*	
H38	0.19420	1.30100	0.76540	0.0675*	
H39	0.19860	1.21030	0.85250	0.0675*	
O29	−0.1365 (4)	1.2557 (3)	0.80333 (12)	0.0480 (4)*	
H40	0.57713	0.71760	0.82517	0.0665*	
H41	−0.26740	1.25380	0.80370	0.0675*	

### 3-[(1R,2S)-2-Azaniumyl-1-hydroxypropyl]phenol (2S,3S)-hydrogen 2,3-dihydroxybutanedioate (I) Geometric parameters (Å, º)

**Table:**

O1—C4	1.429 (3)	H15—C7	1.052 (4)
O1—H22	0.898 (3)	H16—C7	1.100 (4)
O2—C10	1.369 (3)	H17—C7	0.984 (4)
O2—H25	0.946 (3)	H18—C8	1.106 (3)
N3—C5	1.494 (3)	H19—C9	1.101 (3)
N3—H20	1.029 (3)	H20—N3	1.029 (3)
N3—H21	1.002 (3)	H21—N3	1.002 (3)
N3—H40	1.051 (3)	H22—O1	0.898 (3)
C4—O1	1.429 (3)	H23—C11	1.106 (3)
C4—C5	1.533 (3)	H24—C12	1.108 (3)
C4—C6	1.491 (3)	H25—O2	0.946 (3)
C4—H13	1.083 (3)	O26—C33	1.286 (3)
C5—N3	1.494 (3)	O27—C33	1.200 (3)
C5—C4	1.533 (3)	O28—C35	1.241 (3)
C5—C7	1.550 (4)	O30—C32	1.407 (3)
C5—H14	1.100 (3)	O30—H37	0.977 (3)
C6—C4	1.491 (3)	O31—C34	1.406 (3)
C6—C8	1.386 (3)	O31—H36	0.946 (3)
C6—C9	1.366 (3)	C32—O30	1.407 (3)
C7—C5	1.550 (4)	C32—C33	1.553 (3)
C7—H15	1.052 (4)	C32—C34	1.562 (4)
C7—H16	1.100 (4)	C32—H39	1.069 (3)
C7—H17	0.984 (4)	C33—O26	1.286 (3)
C8—C6	1.386 (3)	C33—O27	1.200 (3)
C8—C10	1.374 (3)	C33—C32	1.553 (3)
C8—H18	1.106 (3)	C34—O31	1.406 (3)
C9—C6	1.366 (3)	C34—C32	1.562 (4)
C9—C11	1.384 (3)	C34—C35	1.544 (3)
C9—H19	1.101 (3)	C34—H38	1.101 (4)
C10—O2	1.369 (3)	C35—O28	1.241 (3)
C10—C8	1.374 (3)	C35—C34	1.544 (3)
C10—C12	1.367 (3)	C35—O29	1.270 (3)
C11—C9	1.384 (3)	H36—O31	0.946 (3)
C11—C12	1.391 (3)	H37—O30	0.977 (3)
C11—H23	1.106 (3)	H38—C34	1.101 (4)
C12—C10	1.367 (3)	H39—C32	1.069 (3)
C12—C11	1.391 (3)	O29—C35	1.270 (3)
C12—H24	1.108 (3)	O29—H41	0.942 (3)
H13—C4	1.083 (3)	H40—N3	1.051 (3)
H14—C5	1.100 (3)	H41—O29	0.942 (3)
			
C4—O1—H22	108.8 (3)	C6—C9—H19	123.8 (3)
C10—O2—H25	112.5 (3)	C11—C9—H19	115.8 (3)
C5—N3—H20	107.6 (3)	O2—C10—C8	120.4 (3)
C5—N3—H21	114.6 (3)	O2—C10—C12	118.8 (3)
H20—N3—H21	106.9 (3)	C8—C10—C12	120.85 (19)
C5—N3—H40	110.1 (3)	C9—C11—C12	119.6 (2)
H20—N3—H40	108.3 (3)	C9—C11—H23	122.2 (4)
H21—N3—H40	109.0 (3)	C12—C11—H23	117.8 (4)
O1—C4—C5	108.2 (3)	C10—C12—C11	119.5 (2)
O1—C4—C6	109.0 (3)	C10—C12—H24	119.3 (3)
C5—C4—C6	112.9 (3)	C11—C12—H24	120.8 (4)
O1—C4—H13	106.5 (3)	C32—O30—H37	109.1 (3)
C5—C4—H13	110.1 (3)	C34—O31—H36	112.6 (3)
C6—C4—H13	110.0 (3)	O30—C32—C33	110.8 (3)
N3—C5—C4	111.4 (3)	O30—C32—C34	109.3 (3)
N3—C5—C7	109.8 (3)	C33—C32—C34	108.9 (3)
C4—C5—C7	112.9 (3)	O30—C32—H39	113.6 (3)
N3—C5—H14	101.6 (3)	C33—C32—H39	107.2 (3)
C4—C5—H14	109.7 (3)	C34—C32—H39	107.0 (3)
C7—C5—H14	111.0 (3)	O26—C33—O27	127.8 (3)
C4—C6—C8	120.5 (3)	O26—C33—C32	112.5 (3)
C4—C6—C9	119.6 (3)	O27—C33—C32	119.7 (3)
C8—C6—C9	119.89 (19)	O31—C34—C32	111.8 (3)
C5—C7—H15	108.7 (3)	O31—C34—C35	110.2 (3)
C5—C7—H16	105.5 (3)	C32—C34—C35	110.8 (3)
H15—C7—H16	102.6 (3)	O31—C34—H38	113.6 (3)
C5—C7—H17	112.2 (4)	C32—C34—H38	103.9 (3)
H15—C7—H17	118.7 (4)	C35—C34—H38	106.3 (3)
H16—C7—H17	108.0 (3)	O28—C35—C34	116.5 (3)
C6—C8—C10	119.74 (17)	O28—C35—O29	128.7 (3)
C6—C8—H18	118.3 (3)	C34—C35—O29	114.8 (3)
C10—C8—H18	121.9 (3)	C35—O29—H41	115.8 (3)
C6—C9—C11	120.4 (2)		

### (I_VASP) Crystal data

**Table:**

C_9_H_14_NO_2_^+^·C_4_H_5_O_6_^−^	*b* = 8.42062 Å
*M**_r_* = 317.29	*c* = 24.69000 Å
Orthorhombic, *P*2_1_2_1_2_1_	*V* = 1494.81 Å^3^
*a* = 7.18988 Å	*Z* = 4

### (I_VASP) Data collection

**Table:**

*h* = →	*l* = →
*k* = →	

### (I_VASP) Fractional atomic coordinates and isotropic or equivalent isotropic displacement parameters (Å^2^)

**Table:**

	*x*	*y*	*z*	*B*_iso_*/*B*_eq_	
O1	0.19904	0.79641	0.90895		
O2	0.30367	0.55312	1.09380		
N3	0.43993	0.67441	0.82696		
C4	0.20728	0.62634	0.89994		
C5	0.40094	0.58234	0.87743		
C6	0.16477	0.53659	0.95149		
C7	0.56541	0.59839	0.91583		
C8	0.24506	0.58658	1.00022		
C9	0.04789	0.40382	0.95093		
C10	0.21364	0.50247	1.04809		
C11	0.01340	0.32210	0.99913		
C12	0.09585	0.36959	1.04753		
H13	0.10443	0.59411	0.86857		
H14	0.39033	0.45783	0.86419		
H15	0.55482	0.51329	0.94926		
H16	0.69532	0.57332	0.89398		
H17	0.57543	0.71824	0.93284		
H18	0.33191	0.69278	1.00118		
H19	−0.01707	0.36538	0.91312		
H20	0.45057	0.79766	0.83301		
H21	0.33607	0.65249	0.79796		
H22	0.06961	0.82381	0.91785		
H23	−0.07979	0.22003	0.99895		
H24	0.07184	0.30291	1.08469		
H25	0.23133	0.51970	1.12653		
H40	0.56803	0.63905	0.81033		
O26	0.52321	1.25511	0.80787		
O27	0.53712	0.99571	0.83047		
O28	−0.14693	1.09119	0.73653		
O30	0.16346	0.95661	0.81546		
O31	0.21301	1.10837	0.71535		
C32	0.23428	1.11268	0.81422		
C33	0.44886	1.11891	0.81698		
C34	0.15305	1.18926	0.76289		
C35	−0.05925	1.18289	0.76641		
H36	0.15446	1.00232	0.71438		
H37	0.18695	0.90728	0.85137		
H38	0.19793	1.31362	0.75953		
H39	0.18481	1.18368	0.84927		
O29	−0.13548	1.27646	0.80279		
H41	−0.29187	0125805	0.80531		

### (I_VASP) Hydrogen-bond geometry (Å, º)

**Table:**

*D*—H···*A*	*D*—H	H···*A*	*D*···*A*	*D*—H···*A*
O29—H41···O26^i^	1.14	1.33	2.464	173
O30—H37···O1	0.99	1.70	2.686	169
O31—H36···O29^ii^	0.99	1.95	2.885	156
O31—H36···O28	0.99	2.36	2.644	96
N3—H40···O31^iii^	1.05	1.72	2.762	172
N3—H21···O28^ii^	1.05	1.69	2.718	166
N3—H20···O27	1.05	1.78	2.796	161
N3—H20···O30	1.05	2.50	3.111	116
O2—H25···O27^iv^	1.00	1.76	2.709	157
O1—H22···O2^iv^	0.98	2.19	3.113	155
C5—H14···O26	1.10	2.40	3.364	145
C12—H24···O26	1.09	2.72	3.758	154
C4—H13···O28	1.11	2.61	3.410	128
C9—H19···O29	1.09	2.95	4.033	171

Symmetry codes: (i) *x*−1, *y*, *z*; (ii) −*x*, *y*−1/2, −*z*+3/2; (iii) −*x*+1, *y*+1/2, −*z*+3/2; (iv) *x*−1/2, −*y*+3/2, −*z*+2.
